# The Role of IL-17 in Periodontitis and Its Systemic Connections

**DOI:** 10.3390/ijms262210902

**Published:** 2025-11-10

**Authors:** Tobias Bonsmann, Martyna Mochol, Ewa Bonsmann, Lukasz Jablonowski, Andrzej Pawlik, Joanna Rasławska-Socha, Mariusz Lipski, Małgorzata Mazurek-Mochol

**Affiliations:** 1 Department of Periodontology, Pomeranian Medical University in Szczecin, Powstańców Wlkp 72, 70-111 Szczecin, Poland; 2 Department of Restorative Dentistry, Periodontology and Endodontology, University Medicine Greifswald, Walther-Rathenau-Str. 42a, D-17475 Greifswald, Germany; 3 Department of Physiology, Pomeranian Medical University in Szczecin, Powstańców Wlkp 72, 70-111 Szczecin, Poland; 4 Department of Preclinical Conservative Dentistry and Preclinical Endodontics, Pomeranian Medical University in Szczecin, Powstańców Wlkp 72, 70-111 Szczecin, Poland

**Keywords:** interleukin 17, periodontitis, systemic disease

## Abstract

Interleukin 17 (IL-17) is a crucial mediator at the interface of periodontal dysbiosis and host immunity. This review synthesizes current evidence on IL-17 in periodontitis (PD), its systemic connections, and the role of IL-17 gene variants. Clinical and experimental studies show that IL-17 rises in periodontal disease and is associated with the severity of PD via action on epithelial, stromal and osteoblastic cells to promote chemokine release, neutrophil recruitment, cyclooxygenase 2 and prostaglandin E2 synthesis, RANKL expression, osteoclastogenesis, and matrix metalloproteinase activity. Periodontopathogens *Porphyromonas gingivalis* and *Aggregatibacter actinomycetemcomitans* pre-activate the local inflammation-maintaining Th17 response. There is converging evidence linking IL-17-centered signaling with rheumatoid arthritis, diabetes mellitus, and psoriasis in favor of a shared inflammatory network in barrier tissues and synovium. Despite these associations, IL-17 biology is contextually determined with mucosal defense and bone homeostatic roles that caution against unidimensional explanations. Evidence on IL-17A and IL-17F polymorphisms is still heterogeneous across populations with modest and variable risk associations with PD. Clinically, IL-17 in gingival crevicular fluid, saliva, or serum is a potential monitoring biomarker when utilized along with conventional indices. Therapeutically, periodontal therapy that reduces microbial burden may inhibit IL-17 function, and IL-17-targeted therapy has to balance potential benefit to inflammation and bone resorption against safety in oral tissues. The following research must utilize harmonized case definitions, standardized sampling, and multiethnic cohorts, and it must include multiomics to be able to differentiate between causal and compensatory IL-17 signals.

## 1. Introduction

Periodontitis (PD) is a complex and increasingly common disease, affecting many adults. It is well established that supra and subgingival biofilms provoke an inflammatory and immune response in the body [1]. The hypernym “periodontal diseases” includes all pathological processes that affect the periodontium, gingiva, periodontal ligament, cementum, and alveolar bone. In most diseased patients, periodontal diseases manifest as gingivitis or its irreversible deuteropathy, PD, which is caused by the increased accumulation of dental plaque. Although these account for a large proportion of periodontal diseases, one should not ignore the fact that viral infections, tumors and multiple other insults may lead to destructive processes affecting the periodontium. Bearing this in mind, it also seems quite logical that the cause may range from a simple, unifactorial agent, like a herpes simplex virus, to compound, multifactorial bacteria and host immune system-mediated dysbiosis [2].

Gingivitis is defined as clinical gingival inflammation and is easy to diagnose with the help of numerous indices, including bleeding on probing (BOP) as the gold standard [3]. While gingivitis is known to be fully reversible, it can, if not treated in time, progress to the irreversible state of PD. PD is primarily defined as the pathological loss of periodontal ligament, which is measurable as clinical attachment loss (CAL), and loss of alveolar bone, which is measurable as radiographical bone loss (RBL) [4]. PD is an inflammatory disease. Its primary cause is of bacterial origin. Pathologic bacteria, especially those of Socransky’s red complex (*Porphyromonas gingivalis*, *Tannerella forsythia*, *Treponema denticola*), initiate destructive processes in the periodontium [1]. Both the number of these bacteria and the immune response of the diseased host determine the disease entity. Previous studies have proven that “appropriate” cytokine production results in protective immunity, whereas “inappropriate” cytokine production leads to tissue destruction and disease progression [5]. Several pro-inflammatory cytokines are known to play a pathogenic role in different systemic inflammatory diseases like rheumatoid arthritis (RA). IL-17 is proven to be associated with bone destruction in PD [6].

**Figure 1:**
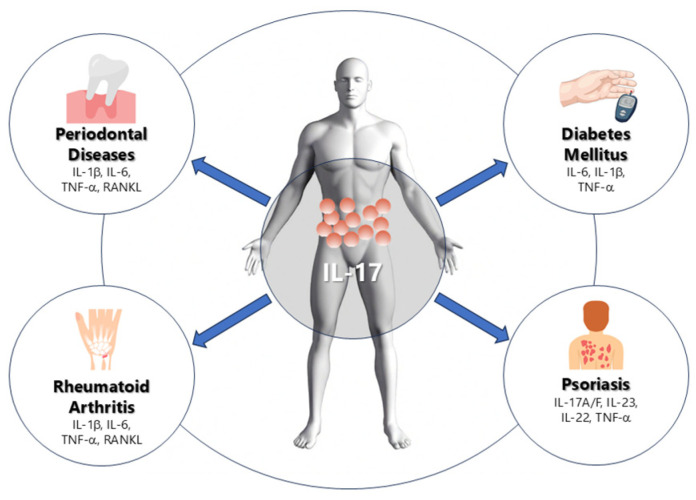
Schematic representation of the role of IL-17 in periodontal and systemic inflammation. IL-17 mediates the relationship between periodontal disease, rheumatoid arthritis, diabetes mellitus, and psoriasis through the induction of pro-inflammatory cytokines such as IL-1β, IL-6, TNF-α, and RANKL, leading to bone resorption, insulin resistance, and chronic inflammation.

**Figure 2:**
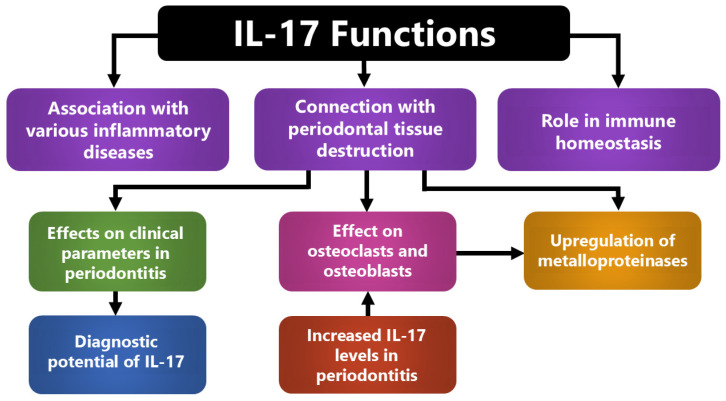
Integrated roles of IL-17 in periodontal inflammation and host–tissue interactions. IL-17 coordinates inflammatory and homeostatic responses by linking immune activation with periodontal tissue destruction. It enhances cytokine and metalloproteinase production, stimulates osteoclast and osteoblast activity, and contributes to systemic inflammatory signaling. These combined actions explain its association with clinical disease severity and its potential as a diagnostic biomarker.

Due to its effect on other systemic diseases, IL-17 and its single nucleotide polymorphisms (SNPs) have been included in medical research concerning the etiology and progression of PD. The elevation or reduction in IL-17 is associated with an alteration in the severity and progression of periodontal diseases, especially of PD [7]. IL-17 is secreted by a variety of innate and adaptive immune cells. After its secretion, inflammatory cascade reactions take place and mediate the initiation and progress of PD and other systemic diseases. Understanding interactions between multiple diseases including PD and, for example, rheumatoid arthritis, psoriasis and diabetes mellitus on a molecular basis (including the role of IL-17—Figure 1) will help dentists and physicians improve their clinical diagnosis and treatment options [7]. Bearing in mind that diagnostic tests that display IL-17 levels (serum, gingival crevicular fluid (GCF)) are nowadays easy and cheap to obtain and given its proven effect on PD at different levels, IL-17 may play an increasingly pivotal role in diagnostics and treatment approaches (Figure 2).

Recent global estimates indicate continued growth in the absolute burden of periodontal diseases through 2040, largely driven by population aging and growth, with persistent regional disparities [8]. Contemporary cytokine syntheses position IL-17 as a nodal mediator of periodontal pathogenesis and a potential therapeutic target that interfaces with precision care [9]. At the same time, emerging work underscores a context-dependent role for IL-17 in mucosal defense and bone homeostasis, which suggests that biomarker use and any IL-17 focused strategies should balance pathogenic and protective functions [10].

This review aims to provide a comprehensive overview of the role of IL-17 in the development of periodontitis and its systemic relevance. It summarizes current knowledge on IL-17 expression and function in periodontal tissues, its contribution to inflammation-driven tissue destruction, and its involvement in the relationship between periodontitis and systemic diseases such as rheumatoid arthritis, diabetes mellitus, and psoriasis. By integrating recent scientific evidence, this review underscores the significance of IL-17 as a potential biomarker and therapeutic target in both periodontal and systemic inflammation.

### Search Strategy and Study Selection

The literature search was conducted in PubMed, Scopus, and Cochrane up to May 2025, using combinations of the following keywords: IL-17, periodontitis, cytokines, systemic inflammation, rheumatoid arthritis, diabetes mellitus, and psoriasis. Reference lists of relevant articles were also screened manually. Screening was performed manually based on title and abstract relevance. Original research studies, reviews, and meta-analyses addressing IL-17 expression, function, or genetic polymorphisms in periodontal and systemic diseases were included. Studies not published in English or lacking relevance to IL-17-mediated mechanisms were excluded. Although no formal quality appraisal tool was applied, study design, sample size, and diagnostic criteria were considered to minimize selection bias. Methodological variability and unadjusted confounders were noted, and a brief overview of these factors is presented in the “Study limitations/notes” column of Table 1.

## 2. IL-17 as Pro-Inflammatory Marker in Periodontitis

IL-17 is a key T-cell derived cytokine with diverse functions in periodontal tissues [24]. It acts on endothelial cells, fibroblasts and epithelial cells, stimulating them to produce chemokines and pro-inflammatory mediators. Among IL-17-producing cells, CD4+ T cells are the most relevant [11].

While IL-17 contributes to host defense and mucosal homeostasis, prolonged production can amplify inflammation and tissue damage [28]. Mitani et al. demonstrated that IL-17 promotes periodontal tissue destruction by inducing receptor activator of NF-κB ligand (RANKL) on CD4+ T cells, thereby enhancing osteoclastogenesis and bone resorption [14]. Similar mechanisms underlie other chronic inflammatory diseases, including rheumatoid arthritis, inflammatory bowel disease, and asthma [12].

Clinical studies consistently show elevated IL-17 in PD. Mitani et al. reported significantly higher IL-17A in gingival crevicular fluid of patients compared with healthy controls [14]. Vernal et al. found a correlation between IL-17 levels and radiographic bone loss, which is mediated by IL-17 induced cyclooxygenase-2 (COX-2), prostaglandin E2 (PGE2) synthesis and RANKL expression in osteoblasts [11,29]. IL-17 also upregulates matrix metalloproteinases, further driving connective tissue breakdown [24]. More pronounced increases are observed in PD, where persistent IL-17 cascades sustain destructive inflammation [14,23,26].

Periodontal pathogens contribute directly to this response. *Porphyromonas gingivalis* and *Aggregatibacter actinomycetemcomitans* stimulate monocytes, enhancing Th17 differentiation and IL-17 production [28]. A recent systematic review confirmed that Th17-related biomarkers, including IL-17, are consistently elevated in PD, supporting their pathogenic role [30].

Isoform specific analyses further refine this picture. Mazurek-Mochol et al. showed that IL-17A expression in gingival tissue correlated with plaque index, whereas IL-17B expression was reduced at the mRNA level in PD patients but correlated positively with CAL, suggesting post-translational regulation [24]. Wankhede et al. similarly reported correlations between IL-17 and CAL and probing depth in aggressive PD [23]. More recently, Altaca et al. demonstrated higher IL-17 levels in gingival crevicular fluid of patients with stage III, IV PD, confirming its association with disease severity [26].

Systematic reviews of the IL-23–IL-17 axis reinforce the diagnostic value of IL-17 in gingival crevicular fluid [29]. Meanwhile, genetic epidemiology offers a more nuanced view: higher genetically predicted IL-17 levels may not uniformly increase PD risk, suggesting potential protective or compensatory roles [31].

When the findings from studies summarized in Table 1 are compared, a general trend emerges: IL-17 concentrations are elevated in the gingival crevicular fluid, saliva, and serum of patients with periodontitis compared to healthy individuals. However, not all reports are consistent. Studies with small sample sizes, variable periodontal classifications, and unadjusted confounders such as smoking or diabetes often failed to detect significant differences. These discrepancies highlight methodological heterogeneity and suggest that IL-17 more accurately reflects local inflammatory burden rather than disease severity itself. Although several studies consistently report elevated IL-17 levels in periodontitis, others have not found statistically significant differences between diseased and healthy sites. Such inconsistencies likely reflect methodological variation, including differences in sample sources (gingival crevicular fluid vs. serum), disease classification criteria, and detection assays. Additionally, biological variability—such as local cytokine compartmentalization or the influence of systemic conditions—may modulate measurable IL-17 concentrations. Together, these factors highlight the importance of standardized protocols and confounder adjustment when interpreting cytokine data in periodontal research.

Overall, IL-17 appears to act as a key modulator at the interface between microbial challenge, host immune response, and periodontal tissue destruction. Its levels in gingival crevicular fluid, saliva and serum correlate with clinical parameters and disease severity, supporting its use as a diagnostic and prognostic biomarker [23,24,26,30,31]. Importantly, recent work also highlights IL-17’s dual nature: it drives destructive osteoclastogenesis yet contributes to mucosal defense and bone homeostasis, underscoring the complexity of IL-17 targeted therapeutic strategies [10].

## 3. Relation Between IL-17 in Periodontitis and Other Systemic Diseases

Systemic diseases, including PD, are linked through shared risk factors and converging inflammatory pathways. Conditions such as diabetes, rheumatoid arthritis, and systemic lupus erythematosus increase susceptibility to destructive periodontal diseases [32]. Although the precise mechanisms are not fully understood, they display overlapping features suggesting common etiological routes [33].

Pro-inflammatory interleukins, particularly IL-17, act as central mediators connecting PD with systemic inflammatory diseases. IL-17 triggers intracellular signaling cascades including NF-κB, MAPK, and JAK/STAT, which stimulate the production of secondary cytokines such as IL-6, TNF-α, and IL-1β. These cascades amplify inflammation both locally in periodontal tissues and systemically, creating feedback loops that exacerbate tissue destruction and contribute to systemic inflammatory burden. Dysregulated IL-17 signaling promotes the recruitment and activation of neutrophils and osteoclasts, linking bone loss in PD with systemic pathologies [7,12]. The shared pathways among PD, autoimmune, and metabolic diseases suggest that IL-17-centered networks serve as a molecular bridge, unifying disparate systemic conditions through common inflammatory mechanisms [34]. Its activity promotes osteoclastogenesis and bone destruction, which are processes that are observed in PD and bone-related systemic diseases such as rheumatoid arthritis [12]. Elevated IL-17 levels have been consistently associated with worse periodontal and systemic outcomes, making it a potential therapeutic target.

Recent work has reinforced these connections. Feng et al. highlighted IL-17A as a central mediator bridging PD with systemic chronic inflammatory diseases, where dysregulated IL-17 signaling contributes to both local tissue breakdown and systemic inflammatory burden [7]. Neurath et al. further demonstrated that cytokines including IL-17 not only sustain gingival inflammation but also disseminate systemically, exacerbating comorbidities and offering potential targets for therapy [9]. Hasan et al. summarized evidence that systemic health is tightly linked to periodontal inflammation with IL-17 acting as one of the pivotal messengers driving this association [34].

Taken together, these findings confirm that IL-17 is not confined to local periodontal pathology but plays a broader role in systemic inflammatory networks. By explicitly connecting intracellular signaling, immune cell recruitment, cytokine amplification, and systemic inflammation, IL-17 demonstrates how PD and systemic diseases are mechanistically intertwined. Its dual involvement underscores the importance of monitoring IL-17 levels in clinical practice and considering periodontal, systemic interactions when designing preventive and therapeutic strategies.

### 3.1. IL-17 and Rheumatoid Arthritis

PD and rheumatoid arthritis are distinct diseases, yet they share inflammatory circuits that canter on dysregulated cytokines, including IL-17. Th17 responses amplify synovitis, osteoclastogenesis and matrix degradation, which mirror tissue breakdown in PD [7,35].

Mechanistically, IL-17 acts on synovial fibroblasts and stromal cells, induces RANKL and matrix metalloproteinases, and cooperates with TNF and IL-6, which accelerates bone erosion and cartilage damage in rheumatoid arthritis [35]. Contemporary tissue atlases of rheumatoid arthritis synovium reveal inflammatory subtypes and cellular neighborhoods that include Th17-related programs and cytokine responsive stromal states, which is a framework that helps explain variable responses to targeted therapy [36].

Clinical and immunologic data support IL-17 as a disease activity signal. Patients with rheumatoid arthritis show expanded pathogenic Th17 and Th22 subsets, and higher IL-17 associates with composite activity scores [37,38]. Systemic links with PD are also evident. Rheumatoid arthritis cohorts have an increased prevalence of periodontal disease, and serum IL-17 can be higher in rheumatoid arthritis patients who also have PD compared with PD alone, which points to additive systemic inflammation [13,39].

Therapeutically, rheumatoid arthritis treatment that reduces IL-17-driven inflammation can coincide with improved periodontal parameters, and periodontal care may attenuate the systemic inflammatory burden in rheumatoid arthritis [7,39]. Single cell profiling under TNF or JAK inhibition shows shifts in pathogenic cell states in synovial fluid, consistent with cytokine pathway modulation, which may include IL-17-linked programs [40]. Overall, IL-17 is a mechanistic bridge between periodontal and synovial pathology, which supports coordinated management that addresses oral inflammation and systemic immune drivers.

### 3.2. IL-17 and Diabetes

Diabetes mellitus is a major chronic inflammatory metabolic disease, and its effects on bone include higher fracture risk and osteolytic lesions that resemble those in PD [41]. Because the two conditions share inflammatory drivers, including Th17 responses, many studies have examined IL-17 as a biomarker to track disease activity and therapy outcomes [41,42].

Mechanistically, diabetes can magnify periodontal inflammation through several routes. Hyperglycemia shifts immune cell programs, increases the Th17 to Treg ratio, and fuels cytokine production, which promotes osteoclastogenesis and connective tissue breakdown in the periodontium [41,42]. Conceptual models of the diabetes–PD axis now explicitly include IL-17 as a contributor to increased periodontal dysbiosis and a heightened host response [42].

Clinical data increasingly support elevated IL-17 in diabetic PD. Inflammatory profiling shows that patients with PD plus type 2 diabetes have stronger systemic and local inflammatory signatures than PD alone with panels that include IL-17 among other mediators [25]. Gingival crevicular fluid studies also report higher IL-17 with concentrations tracking clinical severity [22]. These observations align with a broader framework in which diabetes enhances inflammatory bone loss through IL-17-linked pathways [41].

Genetic and biomarker studies provide a nuanced picture. IL-17A and IL-17F are produced by Th17 cells, and polymorphisms within these loci have been explored. In type 1 diabetes with chronic PD, IL-17A gene variability was associated with immune readouts and microbial patterns, although clinical correlations were limited [16]. Salivary biomarker work in type 2 diabetes and PD suggests that IL-17 can increase with both conditions, sometimes in an additive fashion [18]. The overall message is mixed rather than contradictory. Some cohorts emphasize periodontal status as the primary driver of salivary IL-17, while others show higher IL-17 levels when diabetes and PD coexist [18,22,25].

Taken together, IL-17 sits at the intersection of diabetic inflammation and periodontal tissue destruction. Its measurement in gingival crevicular fluid, saliva, or serum can complement clinical parameters when assessing risk and treatment response in patients with diabetes who also have PD [22,25,41,42].

### 3.3. IL-17 and Psoriasis

Psoriasis is driven by the IL-23–IL-17 axis with IL-17A and IL-17F promoting keratinocyte activation, neutrophil recruitment and sustained cutaneous inflammation [43]. Within this pathway, multiple IL-17 family members, receptors and downstream programs shape disease severity and therapeutic response, which explains the effectiveness of IL-17 and IL-23 inhibitors in clinical practice [43].

Links to periodontal pathology are increasingly recognized. Narrative and systematic overviews report a higher prevalence and severity of PD in psoriatic cohorts along with shared immune features that include elevated IL-17 signaling and related mediators [44,45]. A preclinical model supports bidirectionality, ligature-induced PD aggravated psoriasiform inflammation and, conversely, psoriasiform inflammation worsened periodontal breakdown, which is consistent with a systemic inflammatory loop that features Th17–IL-17 pathways [44]. Clinical studies that profile inflammatory markers further suggest that psoriatic patients show correlations between cytokines, including IL-17, and periodontal indices, reinforcing the concept of a common inflammatory network [46].

Overall, IL-17 functions as a mechanistic bridge between psoriasis and PD. It amplifies epithelial, stromal and myeloid responses in the skin, and it participates in osteoclastogenic, matrix-degrading cascades in periodontal tissues. Monitoring IL-17-related biomarkers and coordinating dermatologic with periodontal care may improve outcomes in patients with coexisting disease [43,44,45,46].

Synthesizing the data summarized in Table 2, patients affected by both periodontitis and systemic inflammatory diseases, such as rheumatoid arthritis, diabetes mellitus, and psoriasis, tend to exhibit higher IL-17 levels than those with either condition alone. This reinforces the concept of a shared inflammatory axis linking oral and systemic immunity. Nevertheless, discrepancies between studies likely arise from variations in systemic disease control, medication regimens, and cytokine detection methods.

## 4. IL-17 Polymorphisms in Periodontitis

Polymorphisms in IL-17A and IL-17F can modulate cytokine expression or function, which may alter susceptibility to PD and influence disease severity. The most studied variants are IL-17A rs2275913 and IL-17F rs763780.

### 4.1. IL-17A rs2275913

Evidence is mixed across populations. A recent systematic review of the IL-23–IL-17 axis catalogued variants in IL-17A–IL-17F–IL-17 receptors and IL-23R, and it reported that only a subset of studies linked rs2275913 to PD with substantial heterogeneity by ancestry and case definitions [47]. A meta-analysis focused on rs2275913 did not show a consistent overall association with chronic PD, although subgroup signals varied and likely reflected sample size and confounding factors such as smoking and diabetes [48]. Primary studies also diverge with some reporting higher risk in A carrying genotypes and others finding null or trend-level effects [15,21,49,50,51].

### 4.2. IL-17F rs763780

Findings are similarly inconsistent. In a well-characterized European cohort, rs763780 and rs2275913 were not associated with PD overall, and stratification by smoking did not reveal robust effects despite small allele frequency shifts [52]. Other case control analyses suggest population-specific links and possible interaction with the subgingival microbiome, but sample sizes are modest and require replication [53,54].

The variability in reported associations across ethnicities likely reflects differences in allele frequencies and linkage disequilibrium structures among populations. Environmental and lifestyle factors such as smoking, glycemic control, and microbial composition may further interact with IL-17 gene variants, modifying their effect on cytokine expression and disease susceptibility. These gene–environment interactions help explain the inconsistent findings between populations and highlight the importance of studying IL-17 genetics in diverse, well-characterized cohorts.

When variants across the IL-23–IL-17 pathway are considered together, the aggregate signal for PD risk is modest. A broad cytokine polymorphism review and meta-analysis concluded that compared with IL-1 family variants, IL-17 polymorphisms show weaker and less consistent relationships with PD, with heterogeneity and possible publication bias that limit clinical translation at present [55]. A cross-disease meta-analysis likewise did not find a uniform effect of IL-17A and IL-17F variants for PD, underscoring the importance of population context and phenotype definition [33].

### 4.3. Clinical Implications

IL-17A and IL-17F genotyping should not be used alone for risk prediction. A pragmatic approach is to consider these variants within a multifactorial matrix together with clinical parameters, environmental exposures such as smoking, and protein level biomarkers—for example, IL-17 in gingival crevicular fluid or saliva. Larger, multiethnic studies with harmonized case definitions and rigorous confounder control are needed to clarify population-specific effects and gene–environment interactions [15,21,33,47,48,49,50,51,52,53,55].

## 5. Discussion

This review summarizes current evidence indicating that IL-17 may play a modulatory role in the interaction between periodontal dysbiosis, immune activation, and tissue destruction. While IL-17 is consistently elevated in periodontal and systemic inflammatory conditions, its causal role remains unconfirmed and requires further mechanistic investigation. Across clinical and experimental studies, IL-17 aligns with periodontal severity and bone loss and sits at the interface between oral and systemic inflammation [7,26,29]. Its biology is dependent on context. IL-17 can support mucosal defense and barrier integrity, while persistent or amplified signaling drives osteoclastogenesis, matrix degradation, and collateral tissue damage [7,56].

Collectively, current findings outline a mechanistic framework in which IL-17 serves as a hub linking microbial dysbiosis to host-mediated tissue injury and systemic immune activation. Through the stimulation of macrophages, fibroblasts, and osteoblasts, IL-17 amplifies IL-1β, IL-6, TNF-α, and RANKL signaling, resulting in osteoclast differentiation and connective-tissue degradation. The same cytokine network propagates inflammatory signals systemically, creating a shared IL-17-driven axis across periodontal and extra-oral diseases. This integrative mechanism is illustrated in Figure 1.

From a biomarker perspective, IL-17 measured in gingival crevicular fluid, saliva, or serum associates with disease presence and activity. Cross-sectional data in stage III and IV PD show higher IL-17 with worse clinical indices, and axis level syntheses confirm consistently elevated IL-17 in periodontal disease compared with health [26,29]. Isoform-specific work adds nuance. In gingival tissue, IL-17A and IL-17B show divergent patterns at mRNA and protein levels with post-translational regulation likely contributing to the observed protein increases in disease [24]. Genetic epidemiology provides a cautious note. An instrumental variable analysis suggested that higher genetically proxied IL-17 is not uniformly associated with greater PD risk, and Mendelian randomization work points to specific circulating immune cell phenotypes as causal drivers of PD, which is a framework that helps separate causal from compensatory IL-17 signals [31,57].

Microbial drivers of the Th17–IL-17 axis remain central. Key periodontopathogens stimulate monocytes and favor Th17 differentiation, which amplifies local cytokine networks and recruits neutrophils, creating a self-reinforcing inflammatory loop [28]. This loop helps explain why IL-17 aligns with connective tissue breakdown and radiographic bone loss through COX-2, PGE2, and RANKL pathways in stromal and osteoblastic compartments [11]. At the molecular level, IL-17 promotes osteoclastogenesis and matrix degradation through the activation of STAT3 and NF-κB signaling pathways in stromal and osteoblastic cells. Engagement of the IL-17 receptor triggers the downstream expression of pro-inflammatory mediators such as IL-6, prostaglandin E2 (PGE2), and RANKL, which collectively enhance osteoclast differentiation and bone resorption [6,7,12]. Moreover, IL-17 acts synergistically with IL-23 and TNF-α to sustain these inflammatory cascades, amplifying tissue destruction in advanced periodontal lesions [7,11,56].

Together, these findings define a cyclical mechanism in which microbial challenge initiates IL-17 signaling, host genetics modulate its intensity, and tissue biomarkers reflect its clinical consequences. Systemic connections are increasingly clear. Reviews integrating oral and systemic data position IL-17 as a mediator that can disseminate inflammatory signals beyond the periodontium, aligning with epidemiologic links to rheumatoid arthritis, diabetes, and psoriasis [9,31]. In rheumatoid arthritis, IL-17 cooperates with TNF and IL-6 to promote synovial fibroblast activation, RANKL upregulation, and erosive damage with synovial tissue atlases identifying Th17-related inflammatory neighborhoods that may modulate treatment response [13,35,36]. In diabetes, hyperglycemia can skew the Th17 to Treg balance and amplify cytokine output, which aligns with higher local and systemic inflammatory signatures in patients who have both diabetes and PD, although cohort findings vary in magnitude and biomarker panels [25,41,42]. In psoriasis, the IL-23–IL-17 axis drives keratinocyte and myeloid activation. Epidemiology and preclinical models support bidirectional aggravation between skin and periodontal inflammation, which is consistent with a shared Th17 program across barrier tissues [43,44,45].

The polymorphism literature is less conclusive. Meta-analyses and axis-level reviews indicate modest and inconsistent associations between IL-17A rs2275913 or IL-17F rs763780 and PD with substantial heterogeneity by ancestry, case definitions, and environmental exposures such as smoking and glycemic control [47,48,51,55]. These data argue against the clinical translation of single-variant genotyping and support composite risk assessment that integrates clinical indices, exposures, and soluble biomarkers.

Therapeutic implications follow from this biology. Periodontal care that reduces microbial load and inflammatory burden should down-tune IL-17-centered pathways locally and possibly systemically. Emerging data suggest that non-surgical periodontal therapy can modulate IL-17 in gingival crevicular fluid, with patterns influenced by smoking status, which supports the use of IL-17 as a treatment responsive biomarker [27]. In systemic disease, IL-17 and IL-23 inhibitors are effective in psoriasis, while effects are variable in rheumatoid arthritis. Any exploration of IL-17-targeted strategies for PD should balance potential benefits for inflammation and bone resorption with risks related to mucosal defense and candidiasis, given safety observations from the dermatologic use of IL-17 blockade [58], and the documented dual roles of IL-17 in experimental models and human observational studies [7,43,56]. The inhibition of IL-17 can impair mucosal immunity, predisposing to mucocutaneous candidiasis and other opportunistic infections. Moreover, IL-17 contributes to normal tissue repair and epithelial barrier maintenance; therefore, its suppression may delay wound healing and weaken host defense at oral and mucosal sites. These observations underline the need for careful evaluation before extending IL-17-targeted therapy to periodontal settings [7,43,56,58].

**Figure 3:**
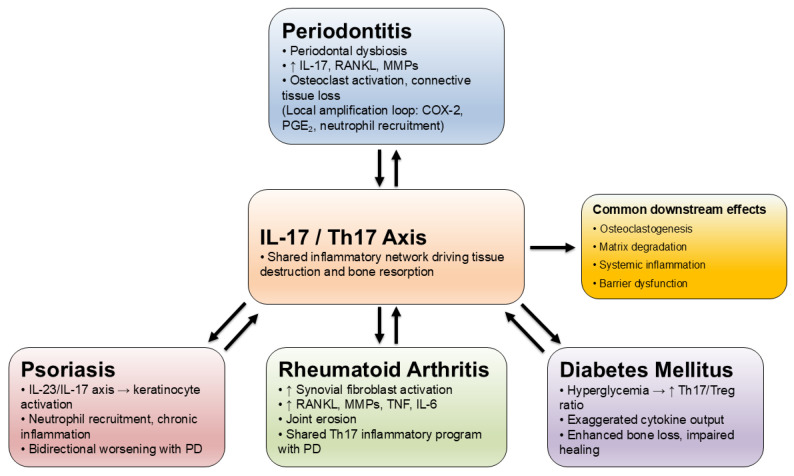
Common IL-17-centered pathways linking periodontitis with systemic diseases.

Collective findings point to a central role of IL-17 in coordinating interactions between microbial, genetic, and immune factors. Through its dual function in promoting local tissue destruction and amplifying systemic inflammation, IL-17 provides a mechanistic link between periodontitis and related chronic inflammatory diseases (Figure 3).

Despite the growing body of evidence linking IL-17 with periodontal and systemic inflammation, current studies are constrained by heterogeneity in design, limited sample sizes, and inconsistent periodontal classification systems. Variations in diagnostic criteria, cytokine detection methods, and the presence of systemic comorbidities such as diabetes or smoking complicate comparisons and may partially explain discrepancies among studies. The absence of uniform adjustment for these confounders further limits the interpretability of IL-17 findings. Future research should address these limitations by adopting harmonized case definitions, standardized sampling and assays, and longitudinal study designs. Multiethnic cohorts with rigorous confounder control and gene–environment interaction testing will be essential to clarify causal mechanisms and to validate IL-17 as a reliable biomarker and therapeutic target. Moreover, most available data are observational, which limits the ability to establish causality between IL-17 expression and disease outcomes. Associations observed in cross-sectional and case-control studies may reflect compensatory immune responses rather than direct pathogenic effects. Longitudinal and interventional studies, ideally integrating multiomics and mechanistic models, are required to determine whether IL-17 acts as a causal driver or as a secondary marker of inflammation. Pragmatically, the most immediate use of IL-17 is as part of a composite monitoring strategy, alongside bleeding on probing, pocket depth, CAL, and radiographic assessment, particularly in patients with comorbid rheumatoid arthritis, diabetes, or psoriasis.

## Figures and Tables

**Table 1:** Summarized characteristics of included studies.

Authors, Year, Country	Participants				Periodontal Evaluation	Purpose	Study Limitations
	Amount		Sex (M—Male, F—Female)	Age			
	Healthy	Patients					
Vernal et al., 2005, Chile [11]	8	16	Healthy: 5 M, 11 F Patients: 5 M, 9 F	Healthy: 36.4 ± 7.9 Patients: 38.4 ± 8.2	A minimum of 5–6 teeth exhibited periodontal probing depths > 5 mm, attachment loss > 3 mm, and extensive bone loss evident on radiographs.	To evaluate IL-17 levels in gingival crevicular fluid and in the cultured supernatants of gingival cells derived from individuals diagnosed with chronic periodontitis.	Small cohort study (n = 24) and single-center recruitment limit statistical power. IL-17 quantification in GCF is prone to intra-individual variability and sampling bias.
Schenkein et al., 2010, USA [12]	67	102	N/A	Healthy: 22.9 ± 0.9 Patients: LAgP: 20.4 ± 1.0 GAgP: 31.2 ± 1.1	A minimum of 8 teeth showed interproximal attachment loss of ≥5 mm with no fewer than 3 of these teeth being other than first molars or incisors. Individuals diagnosed with localized aggressive periodontitis had disease onset before the age of 30, exhibiting ≥ 5 mm interproximal attachment loss confined to the first molars and incisors, and affecting no more than 2 additional teeth.	Measurement of IL-17 serum concentrations in healthy and diseased patients for comparison.	Unequal group sizes and age distribution across LAgP, GAgP, and healthy groups may confound cytokine comparisons. Serum IL-17 reflects systemic rather than local inflammation, potentially masking periodontal specificity.
Gümüş et al., 2013, Turkey [13]	17	49	Healthy: 11 F Patients: 49 F	N/A	Patients with more than 10 teeth suffering from an inflammatory periodontal disease and osteoporosis or rheumatoid arthritis.	To assess the levels of soluble receptor activator of nuclear factor-kappa B ligand, interleukin (IL)-17A, IL-17E, IL-17F, IL-17A/F, and osteoprotegerin in gingival crevicular fluid and serum of women with rheumatoid arthritis, osteoporosis, and systemically healthy controls, all presenting with periodontal disease.	Female-only cohort; heterogeneity due to inclusion of RA and osteoporosis groups. Medication use (anti-inflammatory or anti-resorptive drugs) could suppress cytokine levels, introducing treatment-related bias.
Mitani et al., 2015 Japan [14]	40	86	N/A	N/A	A minimum of six teeth exhibited sites with PPD ≥ 5 mm, CAL ≥ 6 mm, as well as significant bone loss visible on radiographs.	Examination of the production and expression of IL-35, IL-17, and IL-27 in human gingival tissue and gingival crevicular fluid.	No demographic stratification (age, sex, systemic status) reported. Cross-sectional design without longitudinal follow-up. Cytokine detection limited to GCF and tissue—no serum comparison to verify systemic correspondence.
Chaudari et al., 2016, India [15]	35	70	LAgP patients: 19 M 16 F chronic P. patients: 18 M 17 F healthy subjects: 19 M 16 F	Patients: 37.20 ± 4.21 Healthy: 21.23 ± 4.56	Localized aggressive periodontitis was defined as interproximal CAL impacting a minimum of two permanent teeth, including at least one first molar, and affecting no more than two additional teeth besides the first molars and incisors. CP patients had at least 20 natural teeth and a minimum of six periodontal pockets measuring ≥ 5 mm, or CAL of ≥3 mm, along with local factors associated with the destruction of periodontal tissues.	To investigate the association of IL-17 polymorphism (-197G/A) in CP and LAgP.	Limited genetic sample (n = 105) lacks power to detect small effects. No multivariate control for smoking or oral hygiene. Diagnostic criteria for CP and LAgP may overlap, risking misclassification.
Linhartova et al., 2016, Czech [16]	154	407	Healthy: 75 M 79 F Patients: 195 M 212 F	Healthy: 48.5 ± 10.7 Patients: CP: 52.5 ± 9.8 T1DM: 46.4 ± 13.8 T1DM + CP: 49.9 ± 10.6	The determination of PD or non-PD was made using a thorough clinical examination, assessment of medical and dental history, evaluation of tooth mobility, and radiographic examination.	Investigation of IL-17A −197A/G and IL-17F +7488C/T polymorphisms in individuals with T1DM and CP; evaluation of their associations with IL-17 production and the presence of periodontal pathogens.	Potential population stratification not adjusted for. Glycemic control in T1DM participants not standardized. Cross-sectional framework cannot separate cause from effect regarding IL-17 and T1DM comorbidity.
Vahabi et al., 2018, Iran [17]	75	99	Healthy: 29 M 46 F Patients: 56 M 43 F	Patients: 40.28 (mean) Healthy: 31.67 (mean)	In the present study, CP cases with a CAL of ≥5 mm in more than 30% of their remaining teeth were classified as severe CP, those with 3–4 mm CAL as moderate CP, and those with 1–2 mm CAL as mild CP.	To examine the relationship between IL-2 (T-330G), IL-16 (T-295C), and IL-17 (A-7383G) gene variants and the risk of developing CP in an Iranian population.	Small, hospital-based sample. Possible genotyping errors unverified by replication. Environmental or lifestyle confounders (smoking, diet) not accounted for. Only mild/moderate CP cases analyzed, reducing phenotype spectrum.
Saxena et al., 2020, India [18]	17	68	N/A	N/A	Individuals were classified as having CP if they had sites with CAL of ≥4 mm PD of ≥5 mm at four or more teeth per jaw and alveolar bone loss of ≥50% in two or more quadrants.	Investigation of the relationship between salivary levels of developmental endothelial locus-1 and IL-17 in chronic periodontitis.	Small single-center sample; limited control of systemic comorbidities such as diabetes or smoking. Cross-sectional saliva-based design does not establish temporal relationships.
Jiménez et al., 2021, Chile [19]	39	43	Healthy: 21 M 18 F Patients: 18 M 25 F	Individuals with untreated PD: 42.95 (mean) Individuals with untreated psoriasis and untreated PD: 50.2 (mean)	Severe periodontal disease was classified as having at least two interproximal sites with CAL ≥ 6 mm on separate teeth along with at least one interproximal site with probing pocket depth ≥ 5 mm. Moderate PD was classified as having at least two interproximal sites with CAL ≥ 4 mm on different teeth and/or at least two interproximal sites with PPD ≥ 5 mm. Cases were considered no or mild PD if they did not fulfill the criteria for either severe or moderate PD.	To assess the concentrations of IL-17A, IL-22, IL-23, S100A7, S100A8, and S100A9 in gingival crevicular fluid of psoriatic and periodontally healthy individuals, both with and without periodontal disease, and to examine their association with psoriasis severity.	Moderate sample size, psoriasis severity and systemic therapy not controlled for possibly affecting cytokine output. Multiplex cytokine measurements increase multiple-testing risk without correction.
Wu et al., 2022, Taiwan [20]	0	33	Patients: 21 M 12 F	Patients: 48.58 ± 2.07	The prevalence of periodontal disease was 100%, plaque score was 1.10 ± 0.11, gingival score was 1.37 ± 0.12, sites with probing depth around 4 mm were 9.88 ± 1.57%, sites with CAL around 4 mm were 52.15 ± 4.78%, and the mean number of remaining teeth was 25.91 ± 0.66.	To investigate the relationship between psoriasis severity and periodontal tissue destruction in patients with psoriasis.	No healthy or non-psoriatic control group, restricting generalizability. Periodontal diagnosis based on limited indices, lacking radiographic validation. Cannot determine if psoriasis precedes or follows PD changes.
Malvandi et al., 2022, Iran [21]	118	54	Healthy: 42 M 76 F Patients: 27 M 27 F	Patients: 39.89 ± 6.1 Healthy: 39.25 ± 10.7	Presence of CAL measuring ≥ 5 mm and probing pocket depth of ≥4 mm affecting over 30% of the evaluated sites.	To examine the potential association of the IL-17 gene promoter polymorphic site 197 G > A (rs2275913) with generalized severe chronic periodontitis among individuals of Iranian origin.	Small cohort (n = 172) for genetic association. Lack of adjustment for smoking or microbial profile. Findings require replication in independent populations.
Nair et al., 2022, India [22]	30	60	M:F = 45:45	20–50	GI, PPD, CAL.	Quantify GCF IL-17, IL-18, IL-21 across health, gingivitis, CP.	Sample size insufficient for multivariate analysis. Matching of controls not described. Cytokine assessment limited to GCF without confirmation in tissue or serum compartments.
Wankhede et al., 2022, India [23]	30	60	N/A	Average of all participants = 37.5 years. Participants between 20 and 65 years	Group 1 consisted of participants under 35 years of age who had at least six permanent teeth, excluding the incisors and first molars, with probing pocket depths and periodontal attachment levels of ≥5 mm. Group 2 (CP) included participants exhibiting clinical signs of gingival inflammation, with at least six teeth in each jaw, PPD ≥ 4 mm, and PAL ≥ 4 mm.	To show the relation between IL-17 and severity of PD (AgP, CP).	Small, convenience sample with wide age range. Clinical measurements may vary by examiner. No information on systemic health or medications; potential inter-group differences in oral hygiene.
Mazurek-Mochol et al., 2023, Poland [24]	14	14	Healthy: 5 M, 9 F Patients: 5 M, 9 F	Healthy: 54.2 ± 12.5 Patients: 52.8 ± 11.3	Interdental CAL of ≥2 mm was present at two or more non-adjacent teeth, or buccal/lingual CAL ≥ 3 mm; periodontal pockets exceeding 3 mm were found at two or more teeth, with the attachment loss not explained by non-periodontal factors; bleeding on probing was noted at sites with deep probing depths ≥ 5 mm; and radiographs showed a minimum of 15% bone loss.	Investigation of the expression patterns and spatial distribution of IL-17A and IL-17B within gingival tissues of individuals affected by periodontal disease.	Very small (n = 28) exploratory sample. Immunohistochemical quantification semi-quantitative and subjective.
Xu et al., 2023, China [25]	20	80	Healthy: 8 M, 12 F Patients: 40 M, 40 F	Healthy: 33.55 ± 6.98 Patients: 52.23 ± 9.65 (CP) 57.23 ± 11.63 (CP+T2DM)	PPD, bleeding index (BI), HbA1c, CAL, FBG.	Compare serum and GCF inflammatory states in CP with/without T2DM vs. healthy.	Cross-sectional design; glycemic control and antidiabetic therapy non-standardized. Potential ethnic limitation to East Asian population. Serum vs. GCF cytokine comparison may be influenced by sampling time.
Altaca et al., 2024, Turkey [26]	30	30	Healthy: 10 M, 20 F Patients: 18 M, 12 F	Healthy: 28.83 ± 9.95 Patients: 40.93 ± 10.14	Full-mouth, GI, PPD, CAL.	Compare GCF IL-6, IL-17, IL-35 in healthy vs. stage III, IV; correlate with indices.	Modest sample size (n = 60) and stage grouping only (III–IV) without full staging–grading framework. Lack of longitudinal assessment to determine IL-17 modulation post-therapy.
Ezgi et al., 2025, Turkey [27]	18	37	N/A	N/A	Baseline and 4 weeks post-non-surgical periodontal treatment (NSPT).	Evaluate the impact of non-surgical periodontal therapy on salivary and gingival crevicular fluid levels of IL-17 and IL-35 in smokers compared to non-smokers.	Small sample size (n = 55) with short follow-up (4 weeks). Possible assay variability between saliva and GCF samples. No blinding or placebo control; smoking status confounds cytokine changes after NSPT.

Biomarkers—IL-17, IL-18, IL-21, IL-22, IL-23, IL-35, IL-6, RANKL, OPG, PGE2. Methods—ELISA, qPCR, multiplex immunoassay as reported in each study. The following indices were assessed: gingival index (GI), plaque index (PI), bleeding on probing (BOP), periodontal probing depth (PPD), and clinical attachment loss (CAL). Bleeding index (BI), glycated hemoglobin (HbA1c), fasting blood glucose (FBG).

**Table 2:** Summary of results.

Authors, Year, Country	Method of Analysis	Collected Sample	Main Results	Conclusion
Vernal et al., 2005, Chile [11]	ELISA	GCF	1. The concentration of interleukin-17 (IL-17) in the gingival crevicular fluid of patients with periodontitis is markedly elevated compared to that observed in healthy individuals. 2. T lymphocytes contribute to the synthesis of IL-17 within gingival tissues. 3. IL-17 has been shown to induce macrophages to release several pro-inflammatory cytokines, including tumor necrosis factor-alpha (TNF-α) and interleukin-1 beta (IL-1β).	An elevation in IL-17 levels within the gingival crevicular fluid of patients with periodontitis suggests that this cytokine may play a contributory role in the pathogenesis of chronic periodontitis (CP).
Schenkein et al., 2010, USA [12]	ELISA	Serum	1. Serum IL-17 levels were scarcely detectable in periodontally healthy individuals, whereas markedly elevated concentrations were observed in patients with localized and generalized aggressive periodontitis. 2. Multivariate analysis revealed a significant association between IL-17 concentrations and periodontal attachment loss, while no correlation was found with smoking status. 3. Th17-mediated immune responses appear to be a distinctive feature of aggressive periodontitis, suggesting a potential pathogenic role of IL-17 in this condition.	It is plausible that elevated serum IL-17 levels, originating primarily from gingival tissues, may contribute to the propagation of inflammatory responses in sites distant from the oral cavity.
Gümüş et al., 2013, Turkey [13]	ELISA	GCF + Serum	Although the total gingival crevicular fluid levels of albumin, osteoprotegerin (OPG), IL-17A, and IL-17A/F did not differ significantly among the study groups, notable differences were observed in the GCF concentrations of soluble RANKL (sRANKL), OPG, IL-17A, IL-17E, IL-17F, and IL-17A/F. The sRANKL/OPG ratio was markedly higher in the rheumatoid arthritis group compared with the osteoporotic and systemically healthy (SH) groups.	The elevated levels of inflammatory mediators observed in patients with rheumatoid arthritis, despite the prolonged administration of anti-inflammatory therapy, suggest an inherent tendency of these individuals to overproduce such mediators.
Mitani et al., 2015, Japan [14]	ELISA	GCF + gingival tissue	1. Concentrations of IL-35 and IL-17 in gingival crevicular fluid were significantly elevated in patients with periodontal disease compared with periodontally healthy participants. 2. A positive correlation was identified between IL-17 levels and CAL. 3. Expression of IL-17A mRNA was markedly upregulated in inflamed gingival tissues relative to healthy control samples.	Interleukin-35 (IL-35) and interleukin-17 (IL-17), but not interleukin-27 (IL-27), appear to be involved in the pathogenic mechanisms underlying periodontal disease.
Chaudari et al., 2016, India [15]	DNA genotyping	Serum	A statistically significant difference in genotype distribution was observed among patients with chronic periodontitis, localized aggressive periodontitis, and healthy subjects. Additionally, a significant variation in allele frequencies was detected within the chronic periodontitis (CP) group.	Polymorphic variation in the IL-17A gene at the −197A/G locus has been implicated in the development of chronic periodontitis and localized aggressive periodontitis within the Indian population. Possession of the A allele at this site appears to confer an increased genetic predisposition to both forms of periodontal disease.
Linhartova et al., 2016, Czech [16]	Immunohistochemical analysis	Subgingival and blood samples	No significant differences were observed in allele or genotype frequencies between patients with chronic periodontitis and those with both type 1 diabetes mellitus and chronic periodontitis. However, the presence of the A allele at the IL-17A−197 locus was associated with an increased risk of developing type 1 diabetes mellitus. Furthermore, carriers of the A allele among patients with type 1 diabetes mellitus exhibited significantly higher HbA1c levels.	Variations within the IL-17A gene may partially affect the metabolic control of type 1 diabetes mellitus and the prevalence of “red complex” bacteria in patients with chronic periodontitis, including those with diabetes. Moreover, the findings support the functional significance of the IL-17A polymorphism, as individuals carrying the A allele exhibited increased IL-17 secretion.
Vahabi et al., 2018, Iran [17]	DNA genotyping	Serum	No polymorphic variation in IL-2 (T-330G) was identified among the examined patients, as the TT genotype was detected in both study groups. Furthermore, none of the evaluated genotypes or alleles of IL-16 (T-295C) and IL-17 (A-7383G) demonstrated a significant association with chronic periodontitis.	Analysis of the Iranian population revealed no evidence of a relationship between the IL-2 (T-330G), IL-16 (T-295C), or IL-17 (A-7383G) genotypes and the occurrence of chronic periodontitis.
Saxena et al., 2020, India [18]	ELISA	Saliva	1. Levels of developmental endothelial locus-1 (Del-1) and interleukin-17 were correlated with the degree of inflammation in chronic periodontitis, and the coexistence of type 2 diabetes mellitus further intensified disease severity. An inverse relationship between salivary Del-1 and IL-17 concentrations was also observed. 2. The coexistence of type 2 diabetes mellitus and chronic periodontitis was associated with elevated salivary IL-17 levels and a concomitant reduction in Del-1 levels.	An upregulation of salivary IL-17 and a concomitant downregulation of salivary Del-1 were observed with increasing severity of periodontal disease and type 2 diabetes mellitus. Moreover, the coexistence of type 2 diabetes mellitus in patients with chronic periodontitis may exacerbate inflammation-driven periodontal tissue destruction.
Jiménez et al., 2021, Chile [19]	ELISA	GCF	No significant differences were observed among the groups in the concentrations of IL-17A, IL-22, IL-23, or S100A7.	Findings from the analysis of immune components in gingival crevicular fluid suggest no evident immunological association between periodontal disease and psoriasis.
Wu et al., 2022, Taiwan [20]	Immunohistochemical analysis	Saliva	Baseline psoriasis severity showed no significant relationship with salivary concentrations of IL-1β, IL-12, IL-17A, interferon-γ, or tumor necrosis factor-α.	Salivary IL-1β, plaque accumulation, and CAL demonstrated associations with the Psoriasis Area and Severity Index. Greater periodontal impairment corresponded to more extensive psoriatic manifestations. The inflammatory burden within periodontal tissues in psoriasis appears to be modulated by anti-inflammatory medication and smoking habits. IL-17, however, did not exhibit any measurable impact on psoriasis severity.
Malvandi et al., 2022, Iran [21]	DNA genotyping	Serum	Although none of the inheritance models revealed a significant association between periodontal disease risk and genotype distribution, certain patterns were noted. The GG genotype occurred more frequently among periodontally healthy controls, whereas the AG genotype was predominant in patients under the codominant model. Under the overdominant model, the GGAA and AG genotypes were more common in healthy individuals.	The A allele and AG genotype of IL-17 may represent potential contributors to an increased susceptibility to generalized chronic periodontitis. Although noticeable differences in allele and genotype distributions were observed between the groups, these differences did not reach statistical significance.
Nair et al., 2022, India [22]	ELISA	GCF	IL-17, IL-18, IL-21 lowest in health, highest in CP. IL-17 and IL-21 correlated with CAL.	IL-17 in GCF tracks periodontal inflammation and severity
Wankhede et al. 2022, India [23]	Immunohistochemical analysis	GCF	1. A significant positive correlation was observed between IL-17 concentrations and both probing attachment loss and probing pocket depth in aggressive periodontitis. 2. In chronic periodontitis, IL-17 levels correlated positively with probing attachment loss, whereas no significant association was found with probing pocket depth.	IL-17 concentrations in gingival crevicular fluid were elevated in both aggressive and chronic periodontitis compared with periodontally healthy sites with higher levels detected in chronic periodontitis than in aggressive periodontitis.
Mazurek-Mochol et al., 2023, Poland [24]	Immunohistochemical analysis	Gingival tissue	1. Enhanced IL-17 protein expression within gingival tissues of individuals affected by periodontal disease appears to result from post-translational regulatory mechanisms. 2. A direct association was identified between IL-17A expression and the local plaque index. 3. Transcriptional activity of IL-17B was markedly reduced in gingival tissues from patients with periodontal disease compared with those from periodontally healthy controls. 4. Expression of IL-17B at the mRNA level demonstrated a positive correlation with CAL.	Upregulation of IL-17 protein expression in gingival tissues of patients with periodontal disease appears to arise from post-translational modifications.
Xu et al., 2023, China [25]	ELISA	GCF + serum	Gingival crevicular fluid volume, total IL-17 concentration, and the RANKL/OPG ratio were elevated in patients with chronic periodontitis and in those with both chronic periodontitis and type 2 diabetes mellitus compared with periodontally healthy controls. These parameters were also higher in patients with chronic periodontitis and type 2 diabetes mellitus than in those with chronic periodontitis alone and showed a positive correlation with fasting blood glucose levels.	Diabetes amplifies local and systemic inflammation, including GCF IL-17, in PD.
Altaca et al., 2024, Turkey [26]	ELISA	GCF	PD—higher IL-6, IL-17, IL-35 vs. healthy. IL-17 correlated with PD and with GI, PPD, CAL in stage IV.	GCF IL-17 is elevated in severe PD and is associated with disease severity.
Ezgi et al., 2025, Turkey [27]	ELISA	Saliva + GCF	Following non-surgical periodontal therapy, clinical parameters showed improvement. In gingival crevicular fluid, concentrations of IL-17 and IL-35 increased in both groups; however, the total IL-17 content in gingival crevicular fluid decreased exclusively among non-smokers.	NSPT modulates IL-17/IL-35; smoking status influences biomarker dynamics.

## Data Availability

Not applicable.

## Abbreviations

The following abbreviations are used in this manuscript:

AgP	aggressive periodontitis
BI	bleeding index
CAL	clinical attachment loss
COX-2	cyclooxygenase-2
CP	chronic periodontitis
Del-1	developmental endothelial locus-1
F	female
FBG	fasting blood glucose
GAgP	generalized aggressive periodontitis
GCF	gingival crevicular fluid
GI	gingival index
HbA1c	glycated hemoglobin A1c
IL	interleukin
LAgP	localized aggressive periodontitis
M	male
NSPT	non-surgical periodontal treatment
OPG	osteoprotegerin
OPR	osteoporosis
PAL	periodontal attachment level
PASI	psoriasis area and severity index
PD	periodontitis
PGE2	prostaglandin E2
PPD	periodontal probing depth
RA	rheumatoid arthritis
RCT	randomized controlled trial
sRANKL	receptor activator of nuclear factor-kappa B ligand
SH	systemically healthy
T1DM	type 1 diabetes mellitus
T2DM	type 2 diabetes mellitus
